# Genome Sequence of the Early 20th-Century Extreme Halophile *Halobacterium* sp. Strain NRC-34001

**DOI:** 10.1128/mra.01181-21

**Published:** 2022-01-13

**Authors:** Priya DasSarma, Brian P. Anton, Jessie M. Griffith, Karina S. Kunka, Richard J. Roberts, Shiladitya DasSarma

**Affiliations:** a Institute of Marine and Environmental Technology, Department of Microbiology and Immunology, University of Maryland, Baltimore, Maryland, USA; b New England Biolabs, Ipswich, Massachusetts, USA; Portland State University

## Abstract

*Halobacterium* sp. strain NRC-34001 is a red, extremely halophilic archaeon isolated in Canada in 1934. Single-molecule real-time sequencing revealed a 2.3-Mbp genome with a 2-Mbp chromosome and two plasmids (235 kb and 43 kb). The genome encodes all conserved core haloarchaeal groups (cHOGs) and a highly acidic proteome.

## ANNOUNCEMENT

The red haloarchaeon *Halobacterium* sp. strain NRC-34001 was originally isolated as “*Serratia cutirubra*” (subsequently, Pseudomonas/“*Halobacter*”/Halobacterium cutirubrum) from Canadian salted buffalo hide by A. G. Lochhead (1890 to 1980) while investigating damaging red stains on the flesh side of salted hide (“red heat”), similar to fish spoilage (1–5). It was vital to the discovery of classical haloarchaeal traits such as hypotonic lysis, spheroplast formation, high-GC content genome, compartmentalization, and endemic halophage infection, all prior to the classification of *Halobacterium* species as members of the Archaea in the third domain of life (6–10).

The strain in this study, obtained from the American Type Culture Collection (ATCC 33170), was grown in CM^+^ (complete medium plus trace metals) at 37°C with shaking at 220 rpm (New Brunswick Innova 4230 instrument; New Brunswick, NJ, USA), as previously described (11, 12). Cells were lysed by osmotic shock, and nucleic acids were isolated using standard phenol extraction-based methods (12–15).

Single-molecule real-time (SMRT) sequencing was performed using the PacBio Sequel platform (Menlo Park, CA). A SMRTbell sequencing library was prepared from 3.9 μg genomic DNA, without further shearing, using the SMRTbell Express template prep kit 2.0. The library was purified by removing SMRTbell reads of <15 kb using the BluePippin size selection system (Sage Science, Beverly, MA) and sequenced on a single SMRT cell (Sequel Binding kit 3.0, Sequel Sequencing Plate 3.0) with a 20-h collection time. Sequencing reads were filtered and assembled *de novo* using the Microbial Assembly pipeline (SMRT Link 9.0.0.92188), run with default parameters. A total of 28,995 subreads aligned to the draft assembly, with a mean subread length of 15,890 bp, and the polished assembly resolved automatically into three circular contigs, with 200× mean coverage. The GC-rich genome was found to consist of a large circular chromosome (2,012,898 bp; GC content, 67.9%) and 2 plasmids, pHcu_235 (235,323 bp; GC content, 59.8%) and pHcu_43 (42,817 bp, GC content, 57.8%).

Genome annotation was performed at NCBI using the Prokaryotic Genome Annotation Pipeline (PGAP) build 3190, and the taxonomy was determined by NCBI Taxonomy (16, 17). The genome includes 1 rRNA operon and 47 tRNA genes and encodes 2,341 proteins with a calculated mean isoelectric point (pI) value of 4.9, a highly acidic proteome characteristic of haloarchaea (18–20). Based on additional in-house analysis using GeneMark.hmm 2, FeatureExtract, Sequence Manipulation Suite, and EMBOSS (21–24), all 799 conserved core haloarchaeal groups (cHOGs), 6 Orc/Cdc6 proteins, 3 TATA-binding proteins, and 4 TFB proteins (25–28) were found, as well as genes coding for bacteriorhodopsin, halorhodopsin, and sensory rhodopsins 1 and 2 (29, 30). The genome also includes a complete buoyant gas vesicle nanoparticle (GVNP) gene cluster, although the strain has not been shown to produce GVNPs (31, 32).

Methylation patterns were determined using the Base Modification and Motif Analysis application within the same SMRT Link environment using default settings (minimum Qmod score = 100). A single methylated DNA motif was identified (^m4^CTAG, the product of M.Hsp34001I, which is commonly found in *Halobacterium* species) (33).

### Data availability.

The *Halobacterium* sp. NRC-34001 genome sequence has been deposited at GenBank (accession numbers CP085884.1, CP085882.1, CP085883.1). The raw data are available in the NCBI Sequence Read Archive under the accession number SRR16600243 and the BioProject accession number PRJNA412908.

## References

[1] DasSarma P, DasSarma S. 2008. On the origin of prokaryotic “species”: the taxonomy of halophilic Archaea. Saline Syst 4:5. doi:10.1186/1746-1448-4-5. 18485204 PMC2397426

[2] Lochhead AG. 1934. Bacteriological studies on the red discolouration in salted hides. Can J Res 10:275–286. doi:10.1139/cjr34-026.

[3] Lochhead AG. 1943. Notes on the taxonomic position of the red chromogenic halophilic bacteria. J Bacteriol 45:574–575. doi:10.1128/jb.45.6.574-575.1943. 16560669 PMC373780

[4] Anderson H. 1954. The reddening of salted hides and fish. Appl Microbiol 2:64–69. doi:10.1128/am.2.2.64-69.1954. 13149145 PMC1056958

[5] DasSarma P, Klebahn G, Klebahn H. 2010. Translation of Henrich Klebahn’s ‘Damaging agents of the klippfish—a contribution to the knowledge of the salt-loving organisms.’ Saline Syst 6:7. doi:10.1186/1746-1448-6-7. 20569477 PMC2912921

[6] DasSarma P, Capes MD, DasSarma S. 2019. Comparative genomics of Halobacterium strains from diverse locations, p 285–322. *In* Das S, Dash HR (ed), Microbial diversity in the genomic era, 1st ed. Academic Press, Cambridge, MA. doi:10.1016/B978-0-12-814849-5.00017-4.

[7] Kushner DJ, Masson G, Gibbons NE. 1965. Simple method for killing halophilic bacteria in contaminated solar salt. Appl Microbiol 13:288. doi:10.1128/am.13.2.288-288.1965. 14325897 PMC1058239

[8] Wais AC, Kon M, MacDonald RE, Stollar BD. 1975. Salt-dependent bacteriophage infecting *Halobacterium cutirubrum* and *H*. *halobium*. Nature 256:314–315. doi:10.1038/256314a0. 1143331

[9] Joshi JG, Guild WR, Handler P. 1963. The presence of two species of DNA in some halobacteria. J Mol Biol 6:34–38. doi:10.1016/S0022-2836(63)80079-0. 13964964

[10] Moore RL, McCarthy BJ. 1969. Characterization of the deoxyribonucleic acid of various strains of halophilic bacteria. J Bacteriol 99:248–254. doi:10.1128/jb.99.1.248-254.1969. 4979441 PMC249995

[11] Berquist BR, Müller JA, DasSarma S. 2006. Chapter 27. Genetic systems for halophilic archaea, p 649–680. *In* Oren A, Rainey F (ed), Methods in microbiology, vol 35. Elsevier Academic Press, San Diego, CA.

[12] Ng WL, Yang CF, Halladay JT, Arora P, DasSarma S. 1995. Protocol 25. Isolation of genomic and plasmid DNAs from *Halobacterium halobium*, p 179–184. *In* DasSarma S, Fleischmann EM (ed), Archaea, a laboratory manual: halophiles. Cold Spring Harbor Laboratory Press, Cold Spring Harbor, NY.

[13] DasSarma S. 1984. The bacterio-opsin gene in *Halobacterium halobium*: high-frequency inactivation by insertion sequences and structure of the messenger RNA. PhD dissertation. Massachusetts Institute of Technology, Cambridge, MA. https://mit.primo.exlibrisgroup.com/permalink/01MIT_INST/ejdckj/alma990002112390106761.

[14] Fomenkov A, DasSarma P, Kennedy SP, Roberts RJ, DasSarma S. 2021. Complete genome and methylome analysis of the box-shaped halophilic archaeon *Haloarcula sinaiiensis* ATCC 33800. Microbiol Resour Announc 10:e00619-21. doi:10.1128/MRA.00619-21. PMC8388548 34435862

[15] DasSarma P, Anton BP, Ehrenheim HAL, Martínez FL, Guzmán D, Roberts RJ, DasSarma S. 2021. Genome sequence of *Halobacterium* sp. strain BOL4-2 isolated and cultured from Salar de Uyuni. Microbiol Resour Announc 10:e01045-21. doi:10.1128/MRA.01045-21. PMC8638584 34854697

[16] Tatusova T, DiCuccio M, Badretdin A, Chetvernin V, Nawrocki EP, Zaslavsky L, Lomsadze A, Pruitt KD, Borodovsky M, Ostell J. 2016. NCBI Prokaryotic Genome Annotation Pipeline. Nucleic Acids Res 44:6614–6624. doi:10.1093/nar/gkw569. 27342282 PMC5001611

[17] Schoch CL, Ciufo S, Domrachev M, Hotton CL, Kannan S, Khovanskaya R, Leipe D, Mcveigh R, O’Neill K, Robbertse B, Sharma S, Soussov V, Sullivan JP, Sun L, Turner S, Karsch-Mizrachi I. 2020. NCBI taxonomy: a comprehensive update on curation, resources and tools. Database (Oxford) 2020:baaa062. doi:10.1093/database/baaa062. 32761142 PMC7408187

[18] Kozlowski LP. 2016. IPC—Isoelectric Point Calculator. Biol Direct 11:55. doi:10.1186/s13062-016-0159-9. 27769290 PMC5075173

[19] Kennedy SP, Ng WV, Salzberg SL, Hood L, DasSarma S. 2001. Understanding the adaptation of *Halobacterium* species NRC-1 to its extreme environment though computational analysis of its genome sequence. Genome Res 11:1641–1650. doi:10.1101/gr.190201. 11591641 PMC311145

[20] DasSarma S, DasSarma P. 2015. Halophiles and their enzymes: negativity put to good use. Curr Opin Microbiol 25:120–126. doi:10.1016/j.mib.2015.05.009. 26066288 PMC4729366

[21] Besemer J, Borodovsky M. 2005. GeneMark: web software for gene finding in prokaryotes, eukaryotes and viruses. Nucleic Acids Res 33:W451–W454. doi:10.1093/nar/gki487. 15980510 PMC1160247

[22] Wernersson R. 2005. FeatureExtract—extraction of sequence annotation made easy. Nucleic Acids Res 33:W567–W569. doi:10.1093/nar/gki388. 15980537 PMC1160149

[23] Stothard P. 2000. The Sequence Manipulation Suite: JavaScript programs for analyzing and formatting protein and DNA sequences. Biotechniques 28:1102–1104. doi:10.2144/00286ir01. 10868275

[24] Madeira F, Park YM, Lee J, Buso N, Gur T, Madhusoodanan N, Basutkar P, Tivey ARN, Potter SC, Finn RD, Lopez R. 2019. The EMBL-EBI search and sequence analysis tools APIs in 2019. Nucleic Acids Res 47:W636–W641. doi:10.1093/nar/gkz268. 30976793 PMC6602479

[25] Capes MD, DasSarma P, DasSarma S. 2012. The core and unique proteins of haloarchaea. BMC Genomics 13:39. doi:10.1186/1471-2164-13-39. 22272718 PMC3287961

[26] DasSarma S, Capes M, DasSarma P. 2008. Chapter 1: Haloarchaeal megaplasmids, p 3–30. *In* Schwartz E (ed), Microbiology monographs. Springer, Berlin, Germany.

[27] Capes MD, Coker JA, Gessler R, Grinblat-Huse V, DasSarma SL, Jacob CG, Kim J-M, DasSarma P, DasSarma S. 2011. The information transfer system of halophilic archaea. Plasmid 65:77–101. doi:10.1016/j.plasmid.2010.11.005. 21094181

[28] Berquist BR, Soneja J, DasSarma S. 2005. Comparative genomic survey of information transfer systems in two diverse extremely halophilic archaea, *Halobacterium* sp. strain NRC-1 and *Haloarcula marismortui*. *In* Gunde-Cimerman N, Oren A, Plemenitaš A (ed), Adaptation to life at high salt concentrations in archaea, bacteria, and eukarya. Cellular origin, life in extreme habitats and astrobiology, vol 9. Springer, Dordrecht, Netherlands. doi:10.1007/1-4020-3633-7.

[29] DasSarma S, Kennedy SP, Berquist B, Ng W-LV, Baliga NS, Spudich JL, Krebs MP, Eisen JA, Johnson CH, Hood L. 2001. Genomic perspective on the photobiology of *Halobacterium* species NRC-1, a phototrophic, phototactic, and UV-tolerant haloarchaeon. Photosynth Res 70:3–17. doi:10.1023/A:1013879706863. 16228359

[30] DasSarma S, DasSarma P, Laye VJ, Schwieterman EW. 2020. Extremophilic models for astrobiology: haloarchaeal survival strategies and pigments for remote sensing. Extremophiles 24:31–41. doi:10.1007/s00792-019-01126-3. 31463573 PMC6954306

[31] DasSarma S, DasSarma P. 2015. Gas vesicle nanoparticles for antigen display. Vaccines (Basel) 3:686–702. doi:10.3390/vaccines3030686. 26350601 PMC4586473

[32] DasSarma P, DasSarma S. 2021. Gas vesicle nanoparticles, p 1–17. *In* eLS, vol 2. John Wiley & Sons, Hoboken, NJ. doi:10.1002/9780470015902.a0029044.

[33] Roberts RJ, Vincze T, Posfai J, Macelis D. 2015. REBASE—a database for DNA restriction and modification: enzymes, genes and genomes. Nucleic Acids Res 43:D298–D299. doi:10.1093/nar/gku1046. 25378308 PMC4383893

